# Delirium Management in Critical Care: Are We Moving Forward or Still Treading Water?

**DOI:** 10.3390/medsci13040301

**Published:** 2025-12-03

**Authors:** Sergej Marjanovic, Ivana Berisavac, Vladimir Tutus, Stefan Boskovic, Maja Omcikus, Tea Jankovic, Adi Hadzibegovic, Sanja Ratkovic, Jasmina Opacic, Jovana Stanisavljevic

**Affiliations:** 1 Faculty of Medicine, University of Belgrade, 11000 Belgrade, Serbia; sergejmarjanovic01@gmail.com (S.M.); vltutus@yahoo.com (V.T.); jankovictea2002@gmail.com (T.J.); sanjartk@yahoo.com (S.R.); jovanaavramovic@yahoo.com (J.S.); 2 Neurology Clinic, Department of Emergency Neurology, University Clinical Center of Serbia, 11000 Belgrade, Serbia; 3 Department of Cardiac Surgery, University Clinical Center of Serbia, 11000 Belgrade, Serbia; 4 Department of Anesthesiology and Intensive Care Medicine, University Hospital Bonn, 53127 Bonn, Germany; stefan92boskovic@gmail.com; 5 Clinic for Pulmonary Diseases, University Clinical Center of Serbia, 11000 Belgrade, Serbia; 6 Department of Anesthesiology, Emergency Center, University Clinical Center of Serbia, 11000 Belgrade, Serbia

**Keywords:** delirium, critical care, ICU, intensive care unit, diagnostic options, nonpharmacological approaches

## Abstract

Delirium is one of the most common yet most elusive syndromes in the ICU, marked by fluctuating disturbances in awareness and attention arising from complex, multifactorial pathophysiological processes. Despite decades of research and the identification of numerous risk factors, delirium continues to evade full understanding and remains a major therapeutic challenge. Its consequences are profound: higher morbidity and mortality, prolonged ICU and hospital length of stay, and a substantial economic burden of thousands of dollars in excess costs. Beyond being a clinical complication, delirium has become a silent disruptor of modern critical care. This raises an urgent and challenging question: rather than endlessly treating the aftermath of delirium, could the key breakthrough lie in reimagining the ICU itself? Transformative investments in architecture, infrastructure, and human-centered design—together with elevating nonpharmacological strategies alongside pharmacological therapies—may hold the potential to prevent delirium at its roots. In this narrative review, we synthesize current knowledge on the epidemiology, etiology, pathophysiology, diagnosis, prevention, and management of ICU delirium. We highlight how innovative ICU redesign, holistic care approaches, and integrated evidence-based strategies may reshape the fight against delirium, turning the ICU into not just a site of survival but a therapeutic tool in its own right.

## 1. Introduction

Delirium is an acute clinical phenotype characterized by fluctuating alterations in awareness and attention, arising from multifactorial pathophysiological mechanisms [1,2]. Although delirium is among the most common syndromes in Intensive Care Unit (ICU) patients, it remains frequently under-recognized, with incompletely understood etiology and pathophysiology, as well as limited nonpharmacological and pharmacological therapeutic options [3]. Furthermore, delirium is associated with increased morbidity, mortality, prolonged ICU and hospital length of stay (LOS), and elevated healthcare costs [2,3,4,5]. On average, each ICU patient who develops delirium requires nearly 5 additional ICU days, 7 extra hospital days, and generates approximately $4000 higher ICU costs and $6000 higher overall hospital costs compared to patients without delirium [5]. Despite decades of research identifying numerous risk factors and proposing potential pathophysiological mechanisms, delirium remains a persistent challenge and one of the greatest clinical problems in modern critical care. This raises an important and thought-provoking question: could the enormous resources currently devoted to managing delirium be strategically redirected toward transforming the ICU itself? Redesigning architecture, making substantial investments in “ICU infrastructure”, and elevating nonpharmacological interventions to the same level as pharmacological therapies could not only enhance delirium prevention and treatment, but also reshape the concept of critical care—creating an environment that supports patients, reduces stress, and facilitates recovery. In this way, rather than constantly “putting out fires” caused by delirium, we could build the hospital of the future—an ICU that serves as a therapeutic tool in its own right.

Throughout history, the management of delirium in the ICU has long centered on physical restraints and sedative medications. Regrettably, although our knowledge of the syndrome’s complex etiology and pathophysiology has expanded, effective targeted therapies are still lacking, leaving clinicians largely reliant on traditional approaches that remain complex and often suboptimal.

Building on this perspective, our narrative review aims to synthesize current knowledge on the epidemiology, etiology, and pathophysiology, diagnosis, prevention, and various aspects of delirium management in adult general medical-surgical ICUs—including both nonpharmacological and pharmacological strategies—with a particular focus on its clinical implications in intensive care. By integrating these insights, we aim to support ongoing efforts to advance research and improve patient care, highlighting opportunities where innovative ICU design and holistic approaches may complement traditional therapeutic interventions.

## 2. Background

According to the Diagnostic and Statistical Manual of Mental Disorders, Fifth Edition: DSM-5, delirium is an acute, fluctuating disturbance of attention and awareness with cognitive impairment, caused by a medical condition, substance exposure, or toxin, and not explained by preexisting disorders [6]. Following the global implementation of validated diagnostic tools and modifications in ICU practice aimed at minimizing oversedation and immobility, the incidence of delirium in many ICUs has decreased by approximately 25% [7,8]. Nevertheless, the incidence of delirium varies considerably across different ICU patient populations. For example, a recent meta-analysis of 10 studies including 8580 critically ill patients who received extracorporeal membrane oxygenation (ECMO) therapy—each with low risk of bias—reported a pooled prevalence around 40% [9]. However, studies in this specific field are lacking, although the absence or minimal presence of pulsatility during ECMO may influence cerebral perfusion and, in turn, may contribute to the development of delirium.

**Figure 1:**
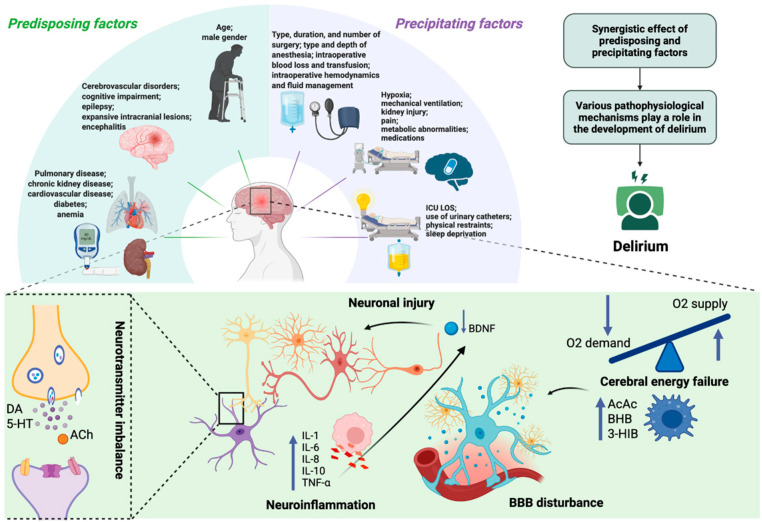
Etiology and pathophysiology of delirium. Delirium results from multiple etiological contributors—including synergistic effects of predisposing (e.g., age, cerebrovascular disorders, chronic kidney disease) and precipitating factors (e.g., infection, mechanical ventilation, hypoxia)—that converge on common pathophysiological mechanisms. These involve neuroinflammation, with elevated pro-inflammatory mediators (e.g., IL-1, IL-6, IL-8, TNF-α) and anti-inflammatory cytokines (e.g., IL-10), neurotransmitter imbalance (e.g., DA, 5-HT, ACh), and neuronal injury (reduced BDNF), together with cerebral energy failure characterized by impaired glucose utilization, accelerated ketogenesis (e.g., AcAc, BHB), and disruption of BBB integrity. ICU LOS—Intensive Care Unit Length of Stay; DA—dopamine; 5-HT—5-hydroxytryptamine (serotonin); ACh—acetylcholine; IL—interleukin; TNF-α—tumor necrosis factor alpha; AcAc—acetoacetate; BHB—β-hydroxybutyrate; 3-HIB—3-hydroxyisobutyric acid; BDNF—brain-derived neurotrophic factor; BBB—blood–brain barrier. Created in BioRender. S, M. (2025) https://BioRender.com/3u7sffo (accessed on 23 October 2025).

Delirium typically arises from the interaction of predisposing factors—such as advanced age, cognitive impairment, and chronic comorbidities—and acute precipitants including type and duration of surgery/anesthesia, infection, hypoxia, metabolic derangements, and drug exposure [10,11,12,13,14,15]. The underlying pathophysiology remains incompletely understood, with proposed mechanisms including blood–brain barrier (BBB) dysfunction, cerebral energy failure [16,17], neuroinflammation [18,19,20], and disruption of neurotransmitter systems [21,22,23,24,25,26]. Systemic inflammatory cytokines (e.g., interleukin-6, tumor necrosis factor-α) can reduce brain-derived neurotrophic factor (BDNF) levels, leading to impaired neuronal function [27]. At the same time, critically ill patients often develop vitamin B6 deficiency due to stress-related metabolic changes [28]. Since vitamin B6 is essential for neurotransmitter synthesis and regulation of the kynurenine pathway, its deficiency may promote the accumulation of neurotoxic metabolites and further suppress BDNF activity. Experimental evidence indicates that vitamin B6 supplementation may redirect tryptophan metabolism toward neuroprotective pathways, which could help maintain BDNF activity and mitigate delirium risk [29]. Despite decades of research, no single hypothesis has been definitively confirmed, and the lack of reliable specific biomarkers continues to limit accurate diagnosis and targeted therapy. The etiology and pathophysiology of delirium are summarized in Figure 1.

## 3. Clinical Subphenotypes of Delirium

Based on current literature, delirium can be classified into several subphenotypes according to different characteristics, reflecting heterogeneity in both clinical presentation and underlying mechanisms. The most widely used classification is based on psychomotor activity—hypoactive, hyperactive, and mixed delirium [30]. Although this scheme has provided a clinically intuitive framework, its reliability and pathophysiological specificity are increasingly questioned. The reported incidence of psychomotor-based subtypes varies widely across studies (40–71% for hypoactive and 12–17% for hyperactive delirium) [31,32], mainly reflecting variations in diagnostic criteria and observer interpretations. Importantly, the predominance of hypoactive delirium highlights a diagnostic gap—its subtle manifestations often escape detection, contributing to worse outcomes. Consequently, there is a growing shift away from purely phenomenological classifications toward biologically driven subphenotypes that could more accurately reflect mechanistic pathways between precipitating and predisposing factors.

Another precipitant-based division of delirium subphenotypes includes septic, hypoxic, metabolic, sedative-associated, and unclassified delirium. Among these, sedative-associated delirium appears most frequently, and prolonged episodes of delirium have been linked to poorer cognitive performance one year later [33]. However, these categorizations, while clinically pragmatic, remain descriptive rather than mechanistic and do not necessarily reflect distinct biological processes. This emphasizes the current limitation of subphenotypic models in predicting outcomes or guiding targeted interventions.

**Figure 2:**
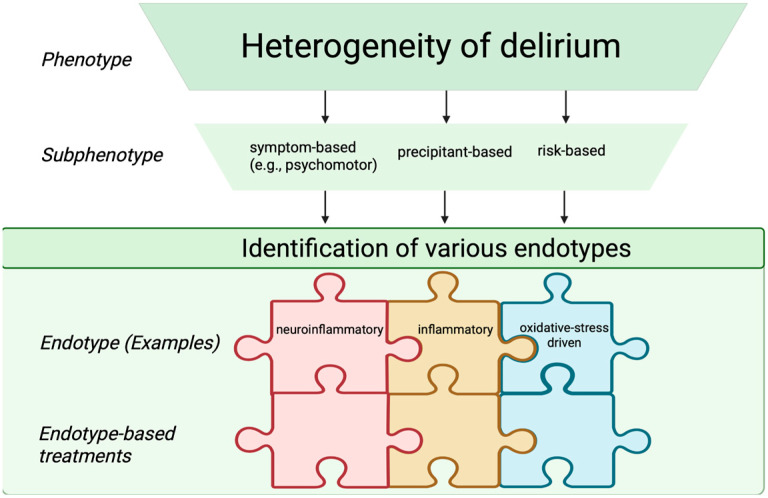
Schematic representation of delirium subphenotypes and endotypes. The endotype-based treatments are depicted as interlocking puzzle pieces, reflecting targeted therapeutic strategies aligned with specific endotypes. Created in BioRender. S, M. (2025) https://BioRender.com/3u7sffo (accessed on 23 October 2025).

Looking forward, the field is moving toward the identification of delirium endotypes—defined as distinct biological mechanisms of disease that may predict treatment responses, mortality risk, clinical course, or therapeutic responsiveness [34]. Although true endotypes cannot yet be established due to the incomplete understanding of delirium pathophysiology, future integrative models may distinguish infection-related, neuroinflammatory, non-inflammatory, and oxidative stress–driven endotypes. Achieving such biological granularity will require multimodal, translational approaches that integrate clinical data with molecular, electrophysiological, and neuroimaging measures to bridge the current gap between syndrome description and pathobiological definition. Although these classifications are not yet implemented in routine clinical practice, recognizing delirium subphenotypes and endotypes may facilitate future individualized interventions, risk stratification, and the design of targeted clinical trials. Figure 2 shows clinical subphenotypes of delirium.

## 4. Diagnostic Options

### 4.1. Confusion Assessment Method for the Intensive Care Unit (CAM-ICU) and the Intensive Care Delirium Screening Checklist (ICDSC)

Delirium in the ICU remains both highly prevalent and substantially underrecognized [3,9,31,32,35]. The most widely used tools for the diagnosis of delirium are the Confusion Assessment Method for the Intensive Care Unit (CAM-ICU) [7] and the Intensive Care Delirium Screening Checklist (ICDSC) [36]. Delirium screening tools are simple to use and typically require only 1–2 min to complete, making them feasible for routine assessment. These screening tools should ideally be used at least twice daily throughout the patient’s entire ICU stay. The CAM-ICU represents a simplified adaptation of the Confusion Assessment Method (CAM) [37], specifically tailored for use in both verbal and non-verbal patients in the ICU. The CAM-ICU tool assesses delirium using four criteria: (1) acute change or fluctuating course of mental status, (2) inattention, (3) altered mental level of consciousness assessed using the current Richmond Agitation-Sedation Scale (RASS), and (4) disorganized thinking. Compared with the reference standard for diagnosing delirium, 2 study nurses using the CAM-ICU had sensitivities of 100% and 93%, specificities of 98% and 100%, and high interrater reliability (kappa = 0.96; 95% confidence interval, 0.92–0.99). The ICDSC evaluates eight key domains: (1) altered level of consciousness, (2) inattention, (3) disorientation, (4) hallucinations or psychosis, (5) psychomotor agitation or retardation, (6) inappropriate speech or mood, (7) sleep–wake cycle disturbances, and (8) symptom fluctuation over the preceding 24 h. Receiver operating characteristic (ROC) analysis demonstrated that the ICDSC has an area under the curve (AUC) of 0.90, with a predicted sensitivity of 99% and specificity of 64% [36].

Although both tools are psychometrically robust, their performance is influenced by patient arousal, sedation level, and the training of personnel conducting the assessments. Importantly, hypoactive delirium and fluctuating symptoms remain often missed even when validated tools are applied, highlighting a difference between screening accuracy under research conditions and everyday ICU practice. Current guidelines recommend routine screening of all ICU patients using a validated instrument such as CAM-ICU or ICDSC; however, adherence to this recommendation remains inconsistent across institutions [38,39]. Improving adherence to delirium screening protocols may require systematic educational initiatives, integration of validated tools into electronic medical records with automated prompts, and regular auditing with feedback to clinical teams to sustain consistent practice. Moreover, there is limited comparative data across different ICU subpopulations (e.g., surgical vs. non-surgical, ventilated vs. non-ventilated), making it unclear whether a single screening tool can be universally optimal [40].

In deeply sedated or paralyzed patients, standard delirium tools may not be reliable. When clinically safe, sedation should be lightened to allow daily wake-up tests or spontaneous sedation interruption for proper neurological assessment. If this is not feasible due to clinical instability or required deep sedation, clinicians should document that delirium screening is temporarily not possible and reassess once consciousness improves. This prevents mislabeling patients who are unarousable due to sedation rather than delirium.

In clinical practice, delirium management involves not only routine CAM-ICU or ICDSC assessments but also strategic reduction in sedatives when clinically safe to enhance patient alertness, the targeted use of antipsychotics when indicated, and continued non-pharmacological interventions.

Future efforts should focus on integrating delirium screening with continuous monitoring technologies to enhance sensitivity and enable early detection of subclinical or fluctuating delirium states. Such multimodal approaches could bridge the gap between structured bedside tools and the complex, dynamic neurobiology of delirium.

### 4.2. Predictive Scores for Delirium Onset

In the last five years, numerous ICU delirium-prediction models have been developed, most using similar predictive factors and statistical models. However, inconsistencies in factor assessment, risk of bias, and limited external validation—primarily restricted to the Prediction model for delirium (PRE-DELIRIC) and Early prediction model for delirium (E-PRE-DELIRIC)—have highlighted the need for further research. Current models generally rely on a single time point within the first 24 h of ICU admission, neglecting patient condition fluctuations, which are crucial in delirium pathophysiology. Future efforts should focus on developing dynamic, clinically actionable prediction models that evolve with the patient’s course [41].

### 4.3. Neuroimaging Techniques in Delirium

Neuroimaging is increasingly utilized in delirium research due to technological advances, allowing identification of structural and functional brain changes in ICU patients. A total of 32 studies (total N = 3187; delirium N = 1086) [42] have used modalities such as magnetic resonance imaging (MRI), computed tomography (CT), diffusion tensor imaging (DTI), transcranial Doppler, near-infrared spectroscopy (NIRS), functional-MRI, single photon emission computed tomography (SPECT), proton MRI spectroscopy (MRS), arterial spin-labeling MRI, and 2-fluoro-2-deoxyglucose positron emission tomography (FDG-PET). Delirium has been associated with brain atrophy, reduced total brain volume, white matter hyperintensities (WMH), disrupted functional connectivity, impaired cerebral autoregulation, decreased cerebral blood flow and oxygenation, and glucose hypometabolism, with evidence suggesting long-term neurological changes following ICU delirium. Despite these insights, the application of advanced neuroimaging is limited by methodological challenges, including patient immobility and technical constraints. Further studies are needed to better explore how in-hospital MRI findings, such as WMH, relate to both short- and long-term outcomes in ICU patients with delirium.

### 4.4. Electroencephalography (EEG) in Delirium

Electroencephalography (EEG) demonstrates considerable potential in evaluating neurophysiological changes associated with delirium, with studies consistently showing both qualitative and quantitative changes to some extent during delirium. However, heterogeneity in patient populations, recording protocols, and delirium diagnostic methods limits standardized recommendations for ICU delirium management. Emerging evidence suggests that increased relative delta power and decreased beta power on bi-electrode EEG may serve as reliable markers, indicating that additional research is needed [43]. Future research should focus on comparing EEG methodologies and delirium evolution alongside patient outcomes to identify clinically applicable parameters.

### 4.5. Biomarkers in Delirium

Biomarkers offer a promising option for understanding, diagnosing, and potentially predicting delirium in critically ill patients. The most compelling biomarkers identified across several studies are S100 calcium-binding protein B (S100β) and neurofilament light (NfL) [44,45,46,47,48,49,50,51]. S100β is a calcium-binding protein, predominantly present in astroglial and oligodendroglial cells in the central nervous system (CNS), and indicates glial activation in response to neuroinflammation, ischemia, and metabolic disturbances [44,45,46,47,48]. Patients with elevated S100β levels tended to experience longer durations of delirium, suggesting a link between glial activation and the persistence of delirium [47].

On the other hand, NfL is a structural neuronal protein that is released into the blood in the presence of neuroaxonal damage [49,50,51]. NfL levels are frequently elevated upon ICU admission in critically ill patients and are associated with worse outcomes. Higher NfL concentrations during the first three days correlate with longer durations of delirium or deep sedation. Moreover, elevated day-one NfL predicts prolonged hospital stay and six-month mortality, with an AUC of 0.81 for mortality prediction [49]. Elevated preoperative blood NfL and Glial Fibrillary Acidic Protein (GFAP) levels were independently associated with increased risk of postoperative delirium, with NfL and GFAP predicting postoperative delirium even after adjustment for age, sex, dementia, frailty, and IL-6 [50].

**Figure 3:**
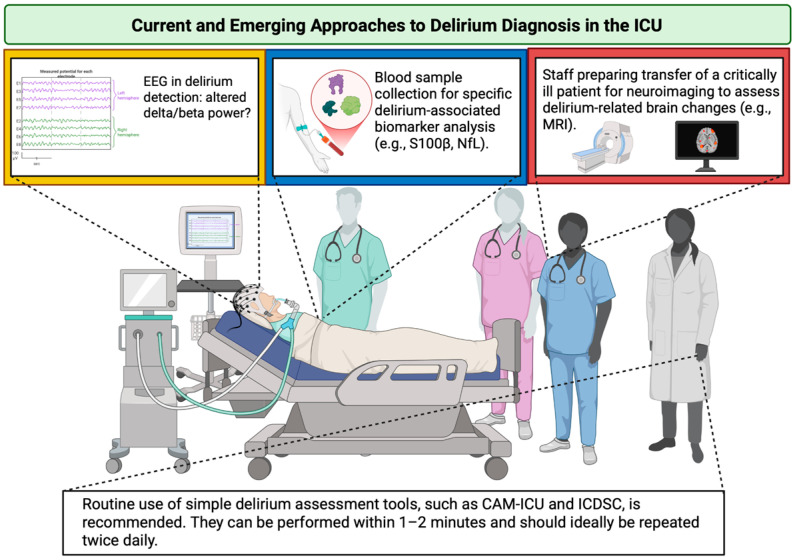
Current and emerging approaches to delirium diagnosis in the ICU. The yellow box emphasizes the need for extensive research on EEG alterations and their incorporation into clinical practice; the blue box denotes the requirement for standardization of delirium-associated biomarkers; the red box illustrates technical and organizational diagnostic constraints in the ICU. ICU—Intensive Care Unit; EEG—electroencephalography; S100β—S100 calcium-binding protein B; NfL—neurofilament light; MRI—magnetic resonance imaging; CAM-ICU—Confusion Assessment Method for the Intensive Care Unit; ICDSC—Intensive Care Delirium Screening Checklist. Created in BioRender. S, M. (2025) https://BioRender.com/3u7sffo (accessed on 23 October 2025).

While biomarker-driven precision medicine holds promise for advancing delirium management, the current evidence base remains fragmented due to a lack of standardization and the absence of clinical protocols. Until robust, reproducible, and clinically actionable biomarkers are validated, bedside clinical judgement must remain central to care. An overview of current and emerging approaches to delirium diagnosis in the ICU is provided in Figure 3.

## 5. Prevention and Treatment Strategies for Delirium

A wide range of strategies exists for the prevention and management of delirium in the ICU, which can be broadly categorized into nonpharmacological and pharmacological approaches. Integrating both approaches is crucial, not only for optimizing patient outcomes but also for advancing best clinical practices, particularly in the context of ICU redesign and holistic care.

### 5.1. Nonpharmacological Approaches

Despite significant advances in delirium research, ongoing updates continue to introduce new strategies aimed at improving survival rates and reducing mortality. Nonpharmacological approaches in the prevention and management of delirium are extremely important and have been recognized and endorsed by the Society of Critical Care Medicine (SCCM) guidelines [40].

#### 5.1.1. Clinical Effectiveness and Cost–Benefit Evidence

**Figure 4:**
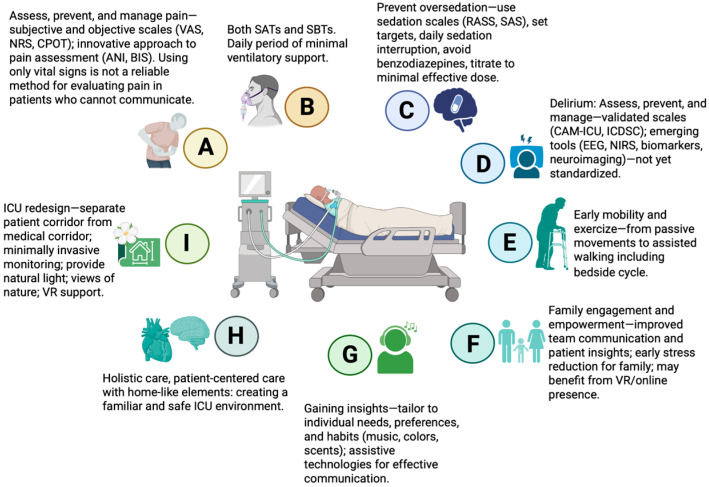
Key elements of the multicomponent A2I bundle in critical care. VAS—Visual Analogue Scale; NRS—Numeric Rating Scale; CPOT—Critical Care Pain Observation Tool; ANI—Analgesia Nociception Index; BIS—Bispectral Index; SATs—spontaneous awakening trials; SBTs—spontaneous breathing trials; RASS—Richmond Agitation-Sedation Scale; SAS—Riker Sedation-Agitation Scale; CAM-ICU—Confusion Assessment Method for the Intensive Care Unit; ICDSC—Intensive Care Delirium Screening Checklist; EEG—electroencephalography; NIRS—near-infrared spectroscopy; VR—virtual reality; ICU—intensive care unit. Created in BioRender. S, M. (2025) https://BioRender.com/3u7sffo (accessed on 23 October 2025).

Various multicomponent bundles are associated with positive outcomes, such as reductions in mortality, ICU LOS, lighter sedation, and the incidence, severity, and duration of delirium in critically ill patients [52,53,54,55]. A well-known multicomponent bundle is the A2F bundle (also referred to as A–F or ABCDEF), comprising: *A*ssess, prevent, and manage pain; *B*oth spontaneous awakening and spontaneous breathing trials; *C*hoice of analgesia and sedation; *D*elirium: assess, prevent, and manage; *E*arly mobility and exercise; and *F*amily engagement and empowerment [56]. Each component is closely interconnected, reflecting the integrated nature of this approach. Two studies have proposed expanding this framework to include additional components, forming the A2I bundle (also referred to as A–I or ABCDEFGHI) [57], or adding R to address *R*espiratory-drive control [58], further enhancing patient-centered care and comprehensive ICU management. The key components of the A2I bundle are summarized in Figure 4.

Recent evidence supports the effectiveness of structured nonpharmacological interventions in critically ill patients. In a propensity score-adjusted analysis, high adherence (≥60%) to the ABCDE bundle significantly reduced inpatient mortality and improved quality-adjusted life-years. However, these benefits were accompanied by increased short-term inpatient costs ($3920) and higher 1-year care costs ($4949). The incremental cost-effectiveness ratios were $15,077 per life saved and $42,120 per quality-adjusted life-year, highlighting that these interventions require substantial resources. Supporting these quantitative findings, studies have shown that delays in patient mobilization and basic physical care—often due to staffing shortages, high patient volume, and frequent admissions and discharges—can deprioritise delirium-focused care. Overall, these findings suggest that while nonpharmacological bundles can improve patient outcomes, their resource-intensive nature and the operational challenges of ICU care highlight the importance of designing an environment that supports delirium prevention, stress reduction, and recovery [59].

#### 5.1.2. ICU Design and Architectural Approaches

The design of a State-of-the-Art ICU, transforming it into a “five-star hotel” experience with spacious, ergonomically designed patient rooms and distinct separation between the medical corridor (for staff) and the visitor corridor (for families), can be seen as part of an effort to redesign the traditional ICU. The goal is to convert from standard “hostile” environment into a more “home-like” setting [60] through thoughtful architectural and interior approaches for fostering a comforting and recovery-oriented environment involve different interventions, such as (1) positioning beds to face windows, and using ambient lighting appropriately to support patients’ circadian rhythms [61]; (2) optimize sensory function—ensure availability and use of glasses and hearing aids while patient is awake [62,63]; (3) cognitive cues—provide calendars, clocks, and family photographs to support orientation and memory while the patient is awake, along with active reorientation to time, place, people, and current situation, which is particularly important in the ICU where the environment is often disorienting and highly stressful [64]; (4) nature exposure—provide access to outdoor or indoor green spaces, such as a garden, patio, or balcony; allow bed positioning toward natural light and fresh air; include plants—hydroponic or potted in protective cases—for visual and sensory stimulation. Although there are currently no studies specifically examining the effect of ICU gardens on delirium, evidence suggests that exposure to gardens can reduce anxiety and promote well-being, highlighting the need for further research in this area [65]; (5) physical environment and safety—create a supportive setting by minimizing noise, including minimizing nocturnal interruptions; ensuring continuity of nursing staff; avoiding unnecessary transfers between beds or units; and permitting personal items from home to provide comfort; promoting sleep through the use of earplugs and eye masks. Physical restraints should be discontinued as early as possible [62,63,66,67]; (6) emerging technologies—immersive virtual reality (VR) interventions that allow ICU patients to virtually interact with family and friends or explore familiar environments through 360° representations of their home and personal spaces represent a potentially promising approach for further study to reduce isolation, stress, and delirium risk. A recent randomized controlled trial (RCT), however, reported similar delirium incidence between the intervention and control groups, while showing improvements in subjective sleep quality [68]. Ongoing RCTs are currently evaluating VR-based interventions for delirium prevention, incorporating immersive sensory stimulation and motion tracking to potentially reduce delirium incidence and improve patient outcomes [69]. Additionally, substantial research is needed to confirm efficacy, optimize protocols, and determine long-term clinical benefits from VR. The use of immersive VR in the ICU has been shown to be feasible even in mechanically ventilated patients, and it can decrease anxiety levels, suggesting that such “digital therapies” may effectively enhance emotional well-being during critical care [70].

In settings where major architectural or environmental redesign is not feasible, effective delirium prevention can still be achieved through strategic engagement of the ICU workforce. Mobilizing a broad team—including anesthesiologists, residents, ICU nurses, respiratory therapists, students, and non-medical staff—ensures that patients receive attentive care and support for orientation, comfort, and basic needs. For example, in one ICU in Serbia, non-medical personnel such as nuns assist patients with feeding and hydration, as well as providing simple aids such as eyeglasses, hearing devices, and headphones. These low-cost interventions enhance sensory input, orientation, and patient comfort, demonstrating that meaningful delirium-preventive strategies can be implemented even in the absence of major infrastructural modifications.

In cardiac surgery patients, a study led by Tutus et al. [71] reported an association between gaseous microemboli (GME) and postoperative delirium. Although this finding is limited to cardiopulmonary bypass, it illustrates the potential impact of iatrogenic factors on cerebral function in critically ill patients. Careful measures to minimize air emboli (e.g., during extracorporeal circulation, hemodialysis, invasive catheter manipulation) may represent an important aspect of delirium prevention in the ICU, although further studies are needed to confirm this association.

**Figure 5:**
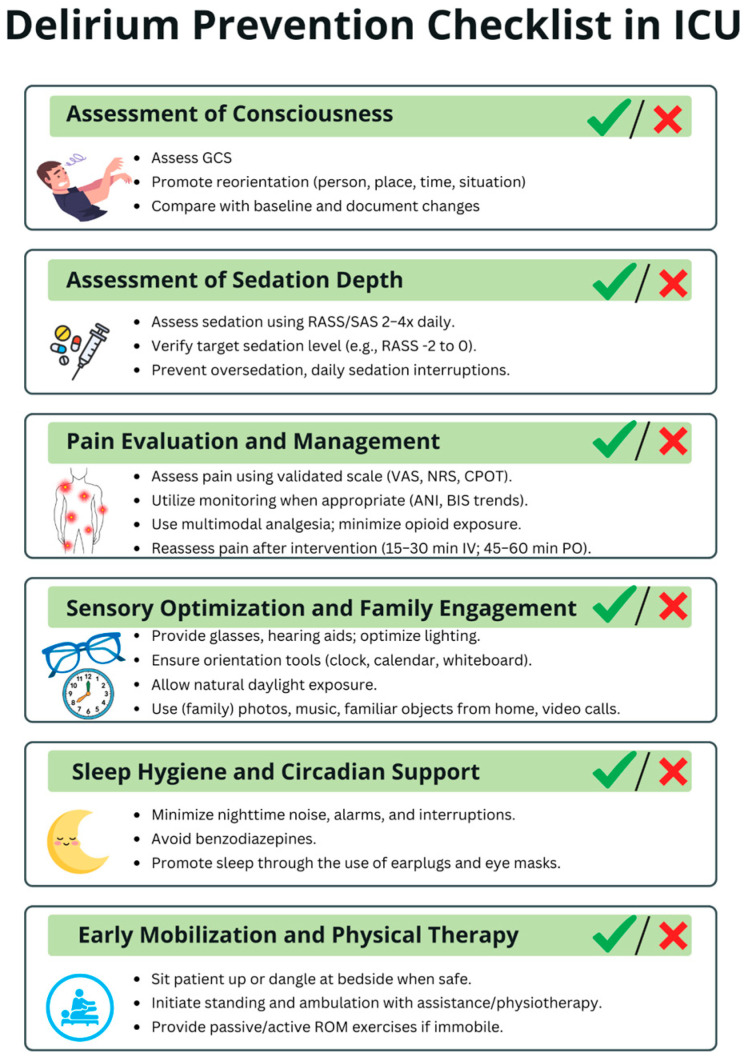
Delirium Prevention Checklist in ICU. This checklist summarizes key daily measures to reduce delirium risk in critically ill patients. The checklist is intended to be used multiple times per day to ensure consistent monitoring and timely implementation of preventive strategies throughout the entire ICU stay. ICU—intensive care unit; GCS—Glasgow Coma Scale; RASS—Richmond Agitation-Sedation Scale; SAS—Riker Sedation-Agitation Scale; VAS—Visual Analogue Scale; NRS—Numeric Rating Scale; CPOT—Critical Care Pain Observation Tool; ANI—Analgesia Nociception Index; BIS—Bispectral Index; IV—intravenous; PO—per os; ROM—range of motion.

Delirium-prevention strategies within the ICU can be viewed hierarchically according to feasibility. Tier 1—easily implementable, low-resource measures include optimizing sensory input (ensuring access to eyeglasses and hearing aids), providing cognitive cues (clocks, calendars, family photos), reducing noise, minimizing nocturnal interruptions, promoting sleep hygiene (earplugs, eye masks), ensuring continuity of nursing staff, avoiding unnecessary bed or unit transfers, and engaging a broad multidisciplinary workforce—including non-medical personnel—to assist with comfort, hydration, and orientation. Tier 2—moderately demanding interventions involve structured reorientation protocols, positioning beds toward natural light, maintaining appropriate ambient lighting to support circadian rhythms, offering controlled exposure to nature (indoor plants, protected greenery), and adopting emerging technologies such as virtual reality, which requires additional equipment, staff training, and protocol development. Tier 3—high-resource, architecture-dependent interventions include comprehensive ICU redesign—such as the creation of spacious rooms, separation of staff and family corridors, improved acoustics, enhanced ventilation, direct access to outdoor or indoor gardens, and construction of recovery-oriented physical environments. To support delirium prevention in the ICU, we developed a Delirium Prevention Checklist, presented in Figure 5.

#### 5.1.3. Staff Knowledge and Delirium

Given the multifactorial nature of delirium, high-quality care in the ICU depends not only on standardized protocols but also on the knowledge and awareness of bedside staff, which may be deficient and has been recognized as one of the significant barriers to effective protocol implementation [72]. Recent work has shown that ICU nurses’ understanding of risk factors, clinical manifestations, and management strategies can be systematically assessed through validated instruments such as the Delirium Knowledge Questionnaire for ICU nurses (DKQ-I), which demonstrated good reliability and validity [73]. These findings emphasize the importance of continuous education and structured training programs to enhance the capacity of the entire ICU team in preventing, recognizing, and managing delirium.

#### 5.1.4. Gut Microbiota and Delirium

Emerging evidence suggests that the composition of the gut microbiota may influence delirium risk in critically ill patients. The presence of *Desulfovibrio* and *Candidatus Soleaferrea* has been associated with an increased risk of delirium, whereas Oxalobacteriaceae, *Holdemania*, *Ruminococcus gnavus*, and *Eggerthella* may be protective. While preliminary, these findings highlight a potential strategy for identifying patients at higher risk and suggest that targeted interventions to optimize gut microbiota—such as tailored enteral nutrition, prebiotics, probiotics, or novel strategies (e.g., fecal microbiota transplantation)—could complement existing nonpharmacological strategies during ICU stay. However, clinical application remains highly experimental, and extensive, well-designed studies are needed to evaluate efficacy, safety, and optimal implementation before these approaches can be recommended for routine use [74].

Effective delirium prevention today relies on consistent implementation of evidence-based nonpharmacological bundles, optimized ICU environments, and improved staff education, all of which can be applied immediately in routine clinical practice.

### 5.2. Pharmacological Approaches

#### 5.2.1. Antipsychotics

Although antipsychotics have long been part of the standard care for patients with delirium, extensive analyses from several RCTs have not demonstrated benefits from their routine use [75,76,77]. For instance, in a randomized, double-blind, placebo-controlled trial, the use of haloperidol or ziprasidone, compared with placebo, had no significant effect on the number of days alive without delirium or coma, 30-day and 90-day survival, time to liberation from mechanical ventilation, or time to ICU and hospital discharge [75]. However, a systematic review and meta-analysis of nine RCTs indicate that antipsychotics may slightly increase the number of delirium-free days and may reduce 28-day and longer-term mortality, although with low certainty of evidence [78]. Reflecting the ongoing uncertainty, the 2025 SCCM Guidelines for the Prevention and Management of Pain, Agitation/Sedation, Delirium, Immobility, and Sleep Disruption in Adult Patients in the ICU (PADIS) panel concluded that they are unable to issue a recommendation for or against the routine use of antipsychotics in adult ICU patients with delirium. These findings contrast with earlier 2018 SCCM PADIS recommendations and highlight the need for further high-quality RCTs to clarify the role of antipsychotics in ICU delirium management [50,78].

Based on current evidence, the management of delirium in the ICU should begin with systematic confirmation of the diagnosis using validated tools such as the CAM-ICU or ICDSC. Once delirium is identified, clinicians should prioritize the identification and correction of contributing factors, including infection, metabolic abnormalities, medication effects, pain, sleep disruption, and environmental stressors. Nonpharmacologic strategies should be the first-line approach. Pharmacologic interventions, including antipsychotics, may be considered only in cases of severe agitation or when nonpharmacologic measures fail. Importantly, any pharmacologic therapy should be individualized, with ongoing reassessment and titration as needed, while research continues to clarify the efficacy and optimal use of antipsychotics in ICU delirium.

#### 5.2.2. Dexmedetomidine

Dexmedetomidine is an α-2A adrenergic receptor agonist, routinely used for sedation, anxiolysis, and analgesia in the ICU [79]. Evidence suggests that dexmedetomidine has anti-inflammatory effects, which may contribute to the prevention of delirium [80]. A recent meta-analysis including nine RCTs demonstrated that dexmedetomidine had a preventive effect on delirium compared with control in elderly patients after orthopedic surgery, supporting its role in delirium prevention [81]. The 2018 SCCM PADIS guidelines suggested the use of dexmedetomidine for delirium in mechanically ventilated adults when agitation was preventing weaning or extubation [40]. In contrast, the 2025 update recommends dexmedetomidine over propofol for sedation in adult ICU patients where light sedation and/or reduction in delirium are prioritized [78]. This reflects a shift from reactive management of agitation to a proactive strategy aimed at optimizing sedation and preventing delirium. However, careful monitoring is necessary upon discontinuation, as studies have shown that high cumulative doses or prolonged infusions of dexmedetomidine may increase the risk of post-sedation delirium, likely due to rebound sympathetic activation [82]. Gradual titration, careful monitoring during withdrawal, and reinforcement of nonpharmacological measures are therefore essential to minimize this risk.

#### 5.2.3. Ketamine

Ketamine is an N-methyl-D-aspartate (NMDA) receptor antagonist with multiple effects on the CNS, including anesthetic, analgesic, and anti-inflammatory actions. The anti-inflammatory properties of ketamine have been documented in several studies [83,84,85]. A recent RCT investigated the effects of continuous intraoperative infusion of S-ketamine in patients undergoing off-pump coronary artery bypass grafting surgery, and demonstrated a statistically significant lower incidence of postoperative delirium in patients who received S-ketamine [85]. However, the 2018 SCCM PADIS guidelines [40] advised against using ketamine for delirium prevention in critically ill adults, and the 2025 update [78] does not provide recommendations for its use in delirium treatment, reflecting the limited evidence and highlighting the need for further high-quality clinical trials.

#### 5.2.4. Melatonin

Melatonin is a serotonin-derived hormone primarily secreted by the pineal gland, and it readily crosses the BBB [86,87]. It is a key regulator of the circadian rhythm [86]. In patients with delirium, sleep–wake disturbances manifest as circadian rhythm inversion, sleep fragmentation, and reduced rapid eye movement (REM) and slow-wave sleep [88]. In sedated, critically ill patients, melatonin levels are significantly lower compared with non-sedated controls and are associated with an increased frequency of delirium in the ICU [89]. A recent meta-analysis of 32 RCTs found that melatonin may reduce delirium incidence, shorten ICU LOS, and improve reported sleep quality, which supports recommendations highlighted for the first time by the 2025 SCCM PADIS guidelines [78,90]. Future investigations are necessary to establish precise protocols (e.g., dosing, timing of administration, quality assurance).

#### 5.2.5. Statins

Statins are widely used drugs with pleiotropic mechanisms of action. Their primary effect is the inhibition of 3-hydroxy-3-methylglutaryl-coenzyme A (HMG-CoA) reductase, the key enzyme in the metabolic pathway of cholesterol synthesis, while they also have important anti-inflammatory and anti-oxidant effects [91]. Although early observational studies suggested that statin therapy might reduce the incidence of postoperative delirium [91,92,93], subsequent systematic review and meta-analysis with high heterogeneity found that statins did not significantly prevent delirium in critically ill or cardiac surgery patients [94]. Reflecting these findings, the 2018 SCCM PADIS guidelines recommend against the routine use of statins for delirium prevention in critically ill adults [40]. Interestingly, a more recent RCT suggested that atorvastatin therapy may be associated with a reduced incidence of postoperative delirium, potentially related to differences in the lipophilicity of specific statins and their ability to cross through the BBB [95]. Nevertheless, this trajectory emphasizes the importance of relying on synthesized evidence from multiple studies, while acknowledging that individual reports may suggest different outcomes, and highlights the need for further high-quality research to clarify the role of statins in delirium prevention.

Key message: Current pharmacologic management should emphasize targeted and evidence-conscious use: antipsychotics are reserved only for short-term control of severe agitation; dexmedetomidine may provide benefit in selected ICU patients, particularly those requiring light sedation; evidence for ketamine remains limited and inconsistent, preventing its current recommendation as standard therapy; while statins and other emerging agents are promising, they remain experimental and require robust clinical trials before integration into routine practice.

## 6. Conclusions and Future Directions

Delirium remains one of the most frequent and challenging complications in critically ill patients, associated with worse short- and long-term outcomes. Despite advances in understanding its pathophysiology and risk factors, effective prevention and treatment strategies remain limited. Current evidence highlights the importance of multimodal, primarily nonpharmacological interventions, such as the A2I bundle.

Future research should focus on refining and standardizing delirium assessment tools, integrating biomarkers and neuroimaging into clinical practice, advancing subphenotyping and endotyping approaches, and implementing emerging technologies such as virtual reality and advanced brain monitoring to enable early detection and individualized interventions. Equally important is the redesign of ICU environments to reduce stress, restore circadian rhythm, and foster human-centered care. Large, multicenter randomized controlled trials are urgently needed to determine the efficacy, cost-effectiveness, and long-term benefits of these strategies.

Ultimately, a paradigm shift is required—viewing the ICU not merely as a site of life support, but as a therapeutic environment designed to preserve cognitive function, promote recovery, and improve the quality of survival for critically ill patients.

## Data Availability

No new data were created or analyzed in this study.
